# Pediatrics: An Evolving Concept for the 21st Century

**DOI:** 10.3390/diagnostics9040201

**Published:** 2019-11-25

**Authors:** Consolato M. Sergi

**Affiliations:** 1 Stollery Children’s Hospital, Department of Laboratory Medicine and Pathology, University of Alberta, Edmonton, AB T6G 2B7, Canada; sergi@ualberta.ca; 2 National “111” Center for Cellular Regulation and Molecular Pharmaceutics, Hubei University of Technology, Wuhan 430068, China

Pediatrics is rapidly evolving, and the diagnostic tools are expanding the spectrum of diagnoses that can be identified at the bedside. The recent progress identified in pediatrics of the last 20 years is astonishing and has consolidated the concept that children are not “smaller adults” and indeed, perinatal and pediatric pathology has become an independent subspecialty in pathology with impressive intersections with fetal medicine, neonatology, and pediatrics. The neonatal intensive care unit (NICU) as well as the pediatric intensive care unit (PICU) play a major role in modern hospitals. These sections of the hospital provide sick newborns and children with the highest level of medical care. Differently from the general medical floors, both units allow intensive nursing care and continuous monitoring of vital parameters, including heart rate, breathing, and blood pressure. The survival of premature babies and small for date newborns has increased exponentially in the last few decades. On the other hand, the immune system, as well as the pulmonary and gastrointestinal organs, remain difficult to manage. At this age an increased rate of infection has been identified, and gastrointestinal dysfunction is common [1,2,3,4,5,6]. Cardiovascular function and sepsis are intimately related and may trigger early death without NICU or PICU intervention [7]. Although the diagnostic procedures in newborns are often overlapping with diagnostic algorithms used at older age, they continue to be a complex and dynamic process which requires a proper investigation of the obstetrical and medical history, skillful physical examination, appropriate laboratory tests, and imaging studies with or without pathological examination of tissue biopsies. On the other hand, even with these steps, diagnosis may remain elusive. The journey from the first appearance of symptom or sign to the final diagnosis may seem sometimes interminable. Today, new techniques seem to shorten this journey swiftly, and next-generation sequencing (NGS) will play a major role in the next decade. NGS is becoming more and more used in clinics other than in academia, although one of the most challenging aspects of NGS testing may reside in its analytical validity. The field of metabolomics is indeed developing at a remarkable rate, particularly in pediatrics. Over the last few years, the pre-clinical detection of pathologies has become more robust and efficient. In electronic libraries, new biomarkers are being identified for several pathologies in neonatology and pediatrics. Management of pediatric diseases may become extenuating, and the use of single nucleotide polymorphisms for the improvement of our approach to some pediatric diagnostic algorithmic failures targeting the interindividual variability may be considered. Pediatric trial networks provide pediatricians, researchers, and agencies with new information on how children may respond to drugs and medications. The present Special Issue collects wet lab research and review articles to highlight some fields of pediatrics that may shape future directions in the diagnosis and management of some diseases. Pediatric heart failure is a challenge in neonatology and pediatrics, and quality assurance criteria are key [7,8,9,10,11]. An open-heart surgery with cardiopulmonary bypass (CPB) remains an interventional procedure accompanied by a high mortality/morbidity rate. Satriano et al. investigated whether blood concentrations of glutathione (GSH), a powerful endogenous antioxidant, changed in the perioperative period [12]. In the perioperative period, the increase in GSH may suggest that a compensatory mechanism to oxidative damage during surgical procedure takes place. The measurement of the interferon (IFN) score has been suggested for the screening of monogenic interferonopathies, like the Aicardi–Goutières syndrome. Moreover, it may be useful to stratify subjects with systemic lupus erythematosus before receiving IFN-targeted treatments. Pin et al. developed an approach to reduce the inter-laboratory variability [13]. These authors provide shared strategies for the IFN signature analysis. They allow different centers to compare data and merge their experiences. Diabetic retinopathy (DR) is a dramatic and major microvascular complication of diabetes mellitus, and very few studies have evidenced the magnitude of this disorder in the pediatric population [14]. The International Society for Pediatric and Adolescent Diabetes (ISPAD) mandates that annual screening for DR should be performed in patients aged 11 years after diabetes of 2 years’ duration and from 9 years of age with diabetes of 5 years’ duration [15]. Kołodziej et al. studied the width of individual retinal layers in patients with type 1 diabetes (T1DM) correlating their data with markers of diabetes metabolic control applying the optical coherence tomography (OCT) study performed using a high definition OCT Cirrus 5000 [16]. The authors found a positive correlation between center thickness and spectral-domain for average glycemia and temporal CT with age at examination, suggesting that selected parameters may be applied as potential markers of preclinical phase of DR in patients with T1DM. Allen and Gupta highlight the current and nearest futuristic perspective of “artificial pancreas” suggesting that soon such a system may not require any manual patient input allowing patients to eat throughout the day without entering any blood sugars or counting carbohydrates [17]. Such a device may be commercially available as technology continues to advance in this direction using artificial intelligence. Sarcoidosis is an inflammatory syndrome of non-necrotizing granulomatous type with multisystemic manifestations, and its occurrence in pediatrics is not an isolated finding any longer. Few cases have been reported of this intriguing disease in children and youth [18]. Chiu et al. revised this topic in detail in this issue [19]. These authors focused on early-onset sarcoidosis, high-risk sarcoidosis, and atypical sarcoid-related diseases. Blau syndrome and early-onset sarcoidosis occur in children younger than five years manifesting with extra-thoracic findings but usually without lymphadenopathy and pulmonary involvement. Endoscopic bronchial ultrasound (EBUS) and transbronchial fine-needle aspiration (TBNA) sampling of intrathoracic lymph nodes and lung may provide good diagnostic yield and excellent patient safety profile in childhood. Respiratory syncytial virus (RSV) bronchiolitis remains an important cause of morbidity in early infancy. RSV belongs to the species of *Orthopneumovirus*. The human RSV (HRSV) infects 60% of infants during their first RSV season, and usually all children show an infection record with this virus by 2–3 years of age [20]. Among the infants infected with RSV, 2–3% will develop bronchiolitis, necessitating hospitalization [21]. RSV bronchiolitis is a major cause of infection and hospitalization in infancy and childhood worldwide. Palivizumab can be employed to prevent this infection in preterm babies, infants with certain congenital heart defects (CHD), infants affected with bronchopulmonary dysplasia (BPD), and infants with congenital malformations of the airway. HRSV bronchiolitis is treated with supportive care, including oxygen therapy, continuous positive airway pressure (CPAP) or nasal high flow oxygen, as required. Rodriguez-Gonzalez et al. identified that left ventricular myocardial dysfunction (LVMD) might occur in healthy infants with HRSV bronchiolitis who develop severe disease and need to be treated at the PICU [22]. N-terminal pro-B-type natriuretic peptide (NT-proBNP) seems to increase the accuracy of traditional clinical markers in predicting the outcomes. Duvekot et al. report a rare event complicating a common adenotonsillectomy. Subcutaneous and mediastinal emphysema followed by group A beta-hemolytic streptococci mediastinitis occurred in a young child, reminding us the life-threatening complications of such surgical procedure [23]. Primary indications for adenotonsillectomy are obstructive sleep apnea (OSA) and recurrent pharyngotonsillitis. Although there is evidence-based medicine supporting the use of such surgical procedures on children affected with OSA that is mainly derived on sleep studies, quality of life, and child behavior, it seems that the impact of surgery on recurrent sore throat symptoms is less well delineated. It has been indicated that children younger than three years and children with OSA of moderate to severe degree, as well as infants affected with significant comorbidities should be admitted for overnight observation. In most patients, simple analgesia is adequate postoperatively, while codeine is contraindicated due to cases of postoperative death as consequence of respiratory suppression. Pain and postoperative hemorrhage (2–4%) are the most common complications, but bleeding can be life-threatening, nevertheless the mortality rate remains small but substantial (1:30,000) [24]. Besides tonsillar enlargement, salivary gland enlargement is a condition which enters in the differential diagnosis of head and neck (H&N) masses. Branchial cysts, sialadenosis, and inflammation of the salivary glands are often seen in childhood and youth, but H&N malignant pathologies also need to be taken into consideration. Sergi et al. critically review the diagnostic features of a pediatric mass of the H&N region [25]. Somatosensory evoked potentials (SSEPs) are crucial in assessing the functional integrity of the neural pathways and for predicting the outcome of perinatal injuries. Barkhuizen et al. studied the translational potential of SSEPs together with sensory function in rodents with perinatal hypoxic-ischemic events [26]. No group differences in the amplitude or latency of the evoked potentials of the preceding sensory response were seen, but nevertheless this method of study is intriguing for the functional recovery. Das and van Landeghem revise the clinicopathological spectrum of bilirubin encephalopathy/kernicterus, which is relatively rare but continues to occur despite universal newborn screening, particularly in middle and low income countries [27]. The authors illustrate the array of clinicopathological findings, and the procedures of diagnostic testing reported to be key in the context of bilirubin encephalopathy and kernicterus. Khan and Sergi report on sialidosis, which is a rare, autosomal recessive inherited disorder, caused by α-N-acetyl neuraminidase deficiency resulting from a mutation in the neuraminidase gene (*NEU1*) and accompanied by cerebral and extra-cerebral manifestations combining the underlying molecular biology, the clinical features, and the morphological patterns of this disorder [28].

Finally, the attention should be drawn to the frontispiece of this book. It is a photograph of a famous sculpture of Horatio Greenough (6 September 1805–18 December 1852). He was an American sculptor who was best known for his two United States government commissions, “The Rescue” and “George Washington”, among others. Before graduating from Harvard, Horatio Greenough sailed to Italy, Rome, to study art. There, the sculptor created many busts. In 1833, he realized “The Ascension of a Child Conducted by an Infant Angel” in marble. The gift to the Museum of Fine Arts in Boston, Massachusetts, United States of America, is a marvelous and touching piece of art and may combine the most elysian characteristics of all Greenough’s sculpture. Both the child and the angel are animated by a deep serenity, which may deeply contrast to the austere Hadrian’s words engraved in the sculpture support (*Animula, vagula, blandula - Hospes comesque corporis - Quae nunc abibis in loca Pallidula, rigida, nudula - Nec, ut soles, dabis iocos*? Little, suave, and wanderer soul, guest and partner of the body - Where are you off to now? Somewhere without color, severe, and empty - Never will you participate in gags as usual). Publius Aelius Hadrianus Augustus was a Roman emperor from 117 to 138 A.D., who died at the age of 62 years following a long and restless reign. Suffering from hypertension and coronary atherosclerosis in the last years of his life, he probably died of congestive heart failure as extensively reported by Dio Cassius and the Historia Augusta records [29]. This suffering has probably shaped some of his scripts. The “Ascension of a Child Conducted by an Infant Angel” conveys an astonishingly gentle, warm, and attractive message of peacefulness, hope, and spirituality in an age of high infant mortality. In 1911, Newmayer wrote that the country which first recognizes its responsibilities to the child would be given the appreciation of the world as being the leading civilized nation [30]. At this time, the United States lagged in the child’s health and welfare, and the infant mortality rate (IMR) positioned the United States as ranked in the 18th position out of 30 countries, with a rate of 135 deaths per 1000 live births. Following the European example, US public health leaders started a national campaign to reduce infant mortality, and, in 1912, the US Children’s Bureau (USCB) was founded (Brosco 1999). Federal and state programs multiplied and, progressively, pediatricians and children’s hospitals also surfaced as the ideal supply of healthcare for children. The improvement of nutrition and treatment of rickets were also crucial and the results of these efforts during the last century have been impressive in all states consolidating the concept of pediatric healthcare in other countries, such as Canada, as well [31]. Digitalization of the imaging in radiology and pathology is a reality in several healthcare institutions worldwide [32,33]. Advances in medical diagnosis and therapy with the implementation of new technologies will be the basis for the future of pediatric healthcare, personalized pediatrics, and quality assurance and controls in the 21st century.
